# Complete Genome Sequence of Nitrogen-Fixing *Paenibacillus* sp. Strain URB8-2, Isolated from the Rhizosphere of Wild Grass

**DOI:** 10.1128/MRA.00814-20

**Published:** 2020-09-03

**Authors:** Kyosuke Kita, Shu Ishikawa, Ken-ichi Yoshida

**Affiliations:** a Department of Science, Technology and Innovation, Kobe University, Kobe, Hyogo, Japan; University of Southern California

## Abstract

We report here the complete genome sequence of nitrogen-fixing *Paenibacillus* sp. strain URB8-2, isolated from the rhizosphere of wild grass in Kobe, Japan, revealing that this bacterium is related to Paenibacillus rhizophilus 7197, a novel species collected recently in Inner Mongolia, China, and that it possesses two gene clusters for distinct types of nitrogenases.

## ANNOUNCEMENT

We suspected that microorganisms with higher nitrogen-fixing capacity might be present in the weed settlements growing on barren sandy soil. Strain URB8-2 was isolated from the rhizosphere of wild grass from the Kobe University campus in Japan, as described previously (1), with some modifications as follows. One gram of the rhizosphere soil was placed in 9 ml of sterile water and mixed thoroughly by vigorous shaking. After the bulk of the soil was precipitated, the aqueous supernatant was heated at 80°C for 10 min and spread onto nitrogen-free agar plates (20 g/liter sucrose, 0.1 g/liter K_2_HPO_4_, 0.4 g/liter KH_2_PO_4_, 0.2 g/liter MgSO_4_·7H_2_O, 0.1 g/liter NaCl, 0.01 g/liter FeCl_3_, 0.005 g/liter Na_2_MoO_4_, and 15 g/liter Difco Noble agar). The plates were incubated at 30°C for 5 days to form transparent slimy colonies. Two colonies were randomly selected and streaked onto the nitrogen-free plates in order to isolate independent colonies. The colony exhibiting the most prominent growth was designated URB8-2.

URB8-2 was grown in tryptic soy broth (TSB) (Becton, Dickinson and Company) at 30°C for 48 h with shaking at 200 rpm. The genomic DNA (gDNA) was isolated using conventional phenol-chloroform extraction (2). The NEBNext Ultra II FS DNA library prep kit for Illumina (New England BioLabs) was used to prepare a gDNA library enriched for DNA fragments around 1,000 bp for Illumina sequencing. Paired-end sequencing (2 × 300 bp) was carried out using the MiSeq platform with the MiSeq reagent kit v3 (Illumina). The raw paired-end reads were trimmed using Sickle v1.33 (3) to remove low-quality (quality [Q] score, <25) reads and short reads (<75 bp). For Nanopore sequencing, another gDNA library was prepared using a rapid barcoding kit (SQK-RBK004) and sequenced on the Nanopore MinION instrument (R9.4 SpotON FLO-MIN106 flow cell). The reads were base called using Albacore v2.2.8 and demultiplexed and trimmed using Porechop v0.2.4 (https://github.com/rrwick/Porechop). Reads with a Q score of <9 and size of <500 bp were discarded using NanoFilt v2.7.0 (4). In total, 2 × 619,857 Illumina reads (genomic coverage, 22×) and 92,346 Nanopore reads (coverage, 60×) with an *N*_50_ value of 5,809 bp were assembled using Unicycler v0.4.8-beta (5), resulting in a single, continuous, circular genome sequence and no plasmid. The circular genome was 5,583,866 bp long with a GC content of 51.6%. Sequence coverage was determined using BBMap v38.86 (https://jgi.doe.gov/data-and-tools/bbtools/), SAMtools v1.9 (6), and minimap2 v2.14-r943-dirty (7). Annotation of the genome using DFAST v1.2.4 (8) identified 5,133 coding sequences, 30 rRNA sequences, and 87 tRNA sequences. The 16S rRNA sequence of URB8-2 was analyzed using the EzBioCloud database (9). Default parameters were used for all software analysis, unless otherwise specified.

The findings suggested that URB8-2 belongs to the genus *Paenibacillus*. The closest strain to URB8-2, sharing 98.89% identity, was *P. rhizophilus* 7197, which is a novel species isolated in Inner Mongolia, China (10). URB8-2 harbors two sets of nitrogenase gene clusters, consistent with other strains of *Paenibacillus* (11).

### Data availability.

The complete genome sequence of *Paenibacillus* sp. strain URB8-2 has been deposited in the DDBJ/ENA/GenBank database under the accession number AP023239. The raw sequence data are available under SRA accession numbers DRX225195 and DRX225196.
